# The effects of splenectomy in murine models of ischemic stroke: a systematic review and meta-analysis

**DOI:** 10.1186/s12974-022-02593-w

**Published:** 2022-09-23

**Authors:** Marko Sternak, Anton Glasnović, Paula Josić, Dominik Romić, Srećko Gajović

**Affiliations:** 1 Croatian Institute for Brain Research, University of Zagreb School of Medicine, Šalata 12, 10000 Zagreb, Croatia; 2 Department of Neurosurgery, University of Zagreb School of Medicine, University Hospital Dubrava, Zagreb, Croatia

**Keywords:** Splenectomy, Ischemic stroke, Monocytes, Inflammation

## Abstract

**Background:**

The spleen, a substantial reservoir of non-differentiated monocytes, may play a crucial role in the pathophysiology of post-ischemic inflammation and influence outcomes after ischemic stroke.

**Aim of the study:**

To analyze splenectomy as a preclinical intervention in murine models of ischemic stroke.

**Methods:**

Following systematic searches of PubMed, Scopus and Web of Science, a qualitative synthesis of study characteristics was performed, and the effect of splenectomy estimated by a three-level random-effects meta-analysis of infarct volumes and a conventional two-level random-effects meta-analysis of neurological deficit scores.

**Results:**

Database searches identified a total of 14 studies, 13 of which were used for meta-analysis. The ischemic lesion volumes were reduced in splenectomized animals compared to the control groups (difference in standardized mean differences: − 1.42; 95% CI [− 1.98, − 0.85]; 95% PI [− 2.03, − 0.80]; *I*^2^_(2)_ = 19.04%; 95% CI [0.00%, 65.49%]; *I*^2^_(3)_ = 47.24%; 95% CI [0.00%, 85.23%]) and neurological deficit scores were improved (− 1.20; 95% CI [− 2.20, − 0.20]; 95% PI [− 4.58, 2.18]; *I*^2^ = 77.5%; 95% CI [50.0%, 89.9%]). A subgroup analysis for infarct volumes showed that splenectomy performed prior to ischemia achieved a higher reduction of the ischemic lesion than when splenectomy was performed immediately prior or after stroke. Although the overall effect size of splenectomy could be classified as large, there was a significant presence of risks of bias, study heterogeneity, and a potential presence of publication bias.

**Conclusion:**

Despite limitations related to heterogeneity, risks of bias, and potential publication bias, this meta-analysis points to the spleen and its functional cell populations as promising targets for the therapeutic modulation of post-stroke inflammation.

**Supplementary Information:**

The online version contains supplementary material available at 10.1186/s12974-022-02593-w.

## Introduction

Stroke remains the second most common cause of death worldwide, and the third most common cause of death and disability combined, with ischemic stroke accounting for 62.4% of all new stroke cases in 2019 [1]. Currently, the only approved therapies for acute ischemic stroke are interventions aimed at re-establishing the cerebral blood flow, including intravenous alteplase administration and mechanical thrombectomy [2]. However, the major drawbacks of these therapies are their very short therapeutic time windows, stringent qualification criteria, and potentially severe side effects. This provides a rationale for further research aimed at establishing new medical interventions, preferably those with neuroprotective and/or neurorestorative effects.

A potential therapeutic target in ischemic stroke is inflammation, which is involved in almost all aspects of post-ischemic damage and repair [3]. Following stroke, the resident microglia are rapidly activated, together with multiple other cell types which simultaneously infiltrate the brain via local blood vessels [4]. One such cell lineage are circulating monocytes/macrophages whose accumulation in the post-ischemic brain was shown to peak at 3–7 days after stroke induction in mice [5]. Additionally, it is currently accepted that mouse monocytes contribute to multiple facets of inflammation and its effects, being represented by two opposing phenotypes: a pro-inflammatory Ly6C^high^ phenotype which is recruited via the C–C chemokine receptor 2 (CCR2), and an anti-inflammatory Ly6C^low^ phenotype, with a high expression of CX3C chemokine receptor 1 (CX3CR1) [6]. Nevertheless, their contribution to stroke damage and subsequent repair is yet to be fully elucidated.

The largest secondary immune organ in the human body, the spleen, represents a substantial reservoir of non-differentiated monocytes, which mobilize to injury sites after ischemia [7]. Therefore, splenectomy could influence the availability of circulating monocytes arriving to the ischemic lesion site and provide evidence of the spleen playing a role in the immune response during the acute period after stroke onset—potentially emerging as a target for various immune therapies that inhibit deleterious responses. A number of preclinical studies combining splenectomy with rodent models of ischemic stroke was performed with varying results; while some have shown that splenectomy prior to stroke induction was associated with reduced infarct size and improved neurobehavioral outcomes [8, 9], others found no significant benefits to spleen removal [10, 11].

The aim of this systematic review and meta-analysis was to evaluate the effects of splenectomy as a preclinical intervention in ischemic stroke and indicate its potential to inform new therapeutic approaches still to be explored. The systematic approach showed that the combined results of selected studies favored splenectomy as a therapeutic intervention, while also presenting a need for better experimental and/or reporting practices in future studies.

## Methods

### Search strategy

The PRISMA guidelines were taken into account during the planning and realization of the systematic review and meta-analysis [12]. Following the methods in a predefined protocol (registered on PROSPERO—CRD42021244723), a search strategy was applied to identify preclinical in vivo studies that used splenectomy as an intervention in murine models of ischemic stroke. Systematic searches of PubMed, Scopus, and Web of Science (All Databases) were performed on January 30, 2021, January 2, 2022, and finally updated on June 28, 2022, using the following search syntax: (splenectomy) AND (mouse OR rat OR mice OR rats OR murine) AND (brain OR neuron OR neurons OR astrocytes OR glia OR neuroglia OR microglia) AND (ischemia OR ischaemia OR ischemic lesion OR stroke). All searches were performed by two investigators independently, with no language or publication date restrictions. Full database searches with reproducible search codes are provided as Additional file 1.

### Eligibility criteria

The studies retrieved by database searches were imported into reference manager software (Mendeley reference manager, Elsevier, NL), where they were first deduplicated using the provided deduplication tool and then manually. The inclusion criteria used to screen titles and abstracts of obtained studies were as follows: (1) the study was a primary research article which produced new and original results; (2) the study was a controlled animal intervention study; (3) it was a preclinical in vivo ischemic stroke study; (4) the population used was adult mice or rats of any sex without comorbidities; (5) the experimental intervention was splenectomy, conducted pre- or post-stroke induction, and (6) the study reported at least one of two predefined primary outcome measures—infarct volume, and/or neurological deficit scores. Excluded were studies without a control group, studies on animals with comorbidities and perinatal/neonatal animals, studies in which animals were subjected to other co-interventions excluding vehicle or saline co-treatment, and studies which did not report the relevant outcome measures. After the title and abstract screening, full texts of resulting studies were reassessed for eligibility using the same criteria. Finally, referenced literature lists of the studies meeting the inclusion criteria were used to identify additional relevant studies. The entire screening process was performed by two investigators independently, and any discrepancies were resolved through discussion or by consultation with the third investigator.

### Data extraction

Relevant data were extracted from all published material, including text and illustrations, in the studies selected by the above procedure. When numerical data were not provided in text, tables or graphs, the authors of included studies were contacted and asked to provide the required data. If no response was obtained after a reminder for the data request, the online graphical tool WebPlotDigitzer was used to extract data from published figures [13]. The following information was collected: author(s), journal and year of publication, animal species, strain, age, sex, stroke model, confirmation of ischemia by blood flow measurement, ischemia duration, splenectomy timing, number of experimental and control groups, number of animals per group, infarct volume and unit of measure, neurological deficit scores and measurement scale type, timing of outcome measurement, and cellular or molecular markers analyzed in populations of interest. All data were extracted by two investigators independently and any discrepancies were resolved through discussion or by consultation with the third investigator (Additional file 2: Table S1 and Additional file 3: Table S2).

### Study risk of bias assessment

Risk of bias was evaluated using the SYRCLE risk of bias tool consisting of the following 10 categories: (1) adequate allocation sequence generation and application; (2) similarity of animal group baseline characteristics; (3) adequate allocation concealment; (4) random housing of animals during the experiment; (5) adequate blinding of caregivers and/or investigators; (6) random selection of animals for outcome assessment; (7) adequate blinding of the outcome assessor; (8) completeness of outcome data for each main outcome; (9) selective outcome reporting, and (10) presence of other sources of bias [14]. Two investigators independently applied the tool to the included studies and assessed the status of each domain (low risk of bias, unclear, high risk of bias) (Additional file 4: Table S3). Any discrepancies were resolved through discussion or by consultation with the third investigator.

### Meta-analysis

From the studies retrieved for systematic review using the previously described methods, eligibility for meta-analysis was assessed under the following requirements: (1) reported group means and their standard deviations (SD) or errors of the mean (SEM) on one of the two predefined primary outcome measures (infarct volume, neurological deficit scores), and (2) reported group sizes. All methods of lesion size assessment, and any neurological deficit scale were included. When studies reported infarct volumes measured in unrelated experimental and control groups, these were regarded as separate experiments and their calculated effect sizes were included as such in the meta-analysis. In cases where multiple methods of assessment were used for structural outcomes measured in the same cohort of animals at the same time point, only the results with the highest control infarct volume were used in the meta-analysis. Furthermore, for functional outcomes measured at > 1 time point, only the last reported time of assessment was used in the analysis. Moreover, if the size of an animal group was reported as a range, the lower value was used. In studies that reported it, SEM was converted into SD using the following formula:

$$\mathrm{SD}=\mathrm{SEM}\times \sqrt{N}.$$

The data were then analyzed using R software [15] under the RStudio graphical environment [16]. For all model fitting and plotting, the “meta” package was used as freely available under the CRAN repository [17]. For each outcome measure, a standardized mean difference (SMD) effect size (Hedges g) was calculated, along with their 95% confidence intervals (CI).

For infarct volumes, to account for the dependence of effect sizes within studies when multiple were included from a single study, a three-level random-effects meta-analysis was conducted; individual effect sizes (level 2, corresponding variance τ^2^_(2)_) were nested within studies (level 3, corresponding variance τ^2^_(3)_). Heterogeneity variance values for each level were calculated using the Restricted maximum-likelihood (REML) estimator and their respective confidence intervals were obtained using the Profile-likelihood (PL) method. Heterogeneity was demonstrated by the Q test and quantified for each level with the *I*^2^_(2)_ and *I*^2^_(3)_ statistics, calculated using the formulas given by Cheung [18]. As *I*^2^_(2)_ and I^2^_(3)_ are both functions of their corresponding variances (τ^2^_(2)_ and τ^2^_(3)_) and the ‘typical’ sampling variance (estimated using the Q statistic), their confidence intervals were obtained using the respective confidence intervals of τ^2^_(2)_ and τ^2^_(3)_. Finally, subgroup analysis was conducted based on splenectomy timing relative to stroke induction, assuming common estimates of τ^2^ due to the small number of studies within subgroups. The amount of heterogeneity explained by the subgroup analysis was quantified for each level by calculating the respective *R*^2^_(2)_ and *R*^2^_(3)_ values [18]. Subgroups were compared using an omnibus Q test of subgroup differences, with additional pairwise comparisons conducted using a Z-test, as described by Borenstein et al. [19].

For neurological deficit scores, because no study reported more than one separate effect size, a conventional two-level random-effects model was used to pool effect sizes, with the heterogeneity variance τ^2^ calculated using the restricted maximum-likelihood (REML) estimator. Heterogeneity was demonstrated by the Q test and quantified using the *I*^2^ statistic. For this analysis, *I*^2^ and its confidence intervals were calculated using Q and the *test-based* method described by Higgins et al. [20].

Additionally, to address the distribution of true effect sizes, 95% prediction intervals (PI) were reported for both meta-analyses.

All statistical tests were reported as significant if *p* < 0.05.

### Publication bias assessment

Publication bias was evaluated using contour-enhanced funnel plots and funnel plot asymmetry was verified using Egger’s regression test.

## Results

### Study selection and data extraction

**Fig. 1:**
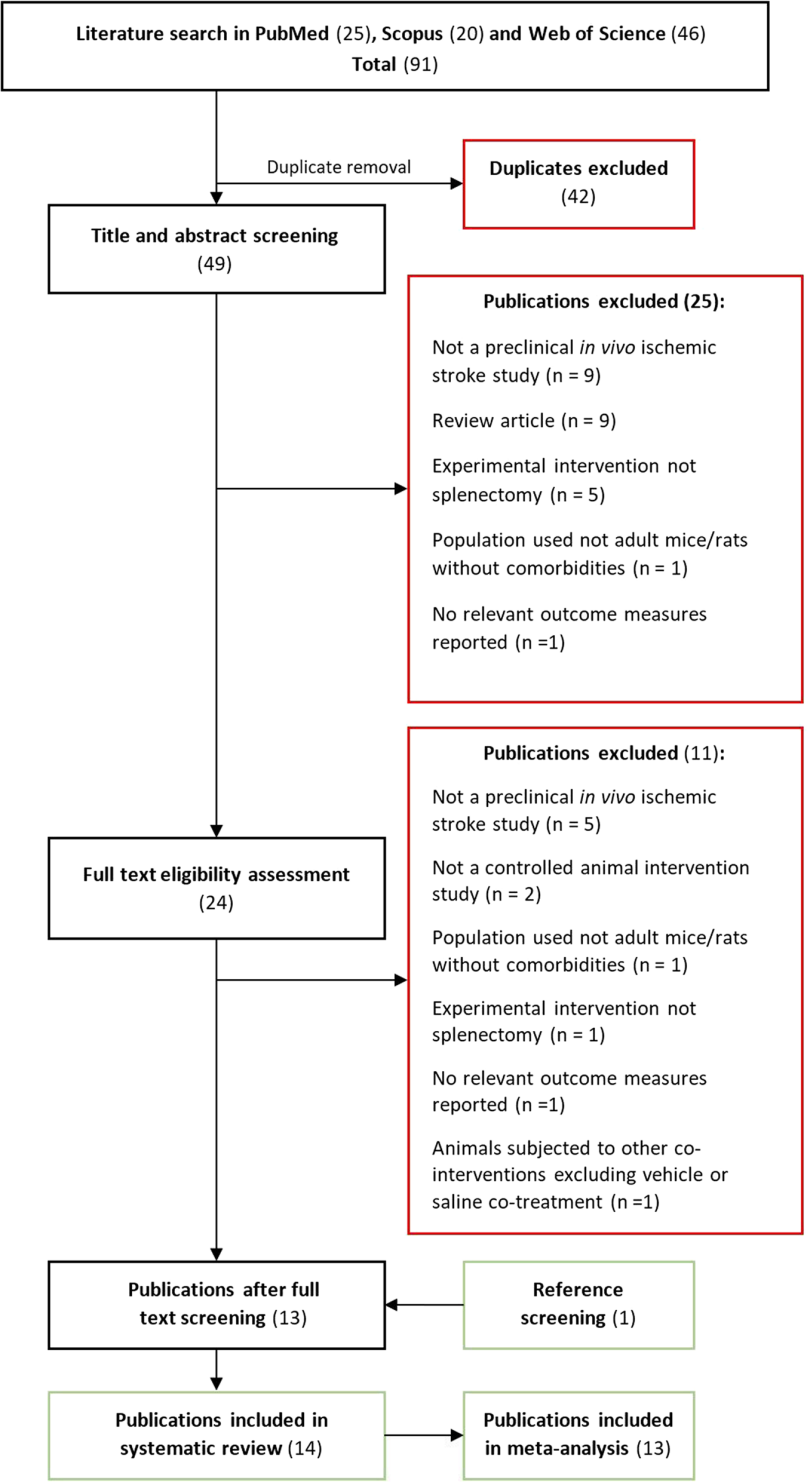
Flowchart of the study selection process. A total of 91 records were retrieved in the systematic search of online databases, last performed on June 28, 2022. After deduplication and application of the inclusion criteria, 14 studies were identified for qualitative synthesis and 13 for meta-analysis

Database searches resulted in a total of 91 identified records (Fig. 1). Following duplicate removal, title, and abstract screening of 49 publications identified 24 studies for further full text reading. Of those 24 studies, 13 matched the established inclusion criteria. One additional study was subsequently identified from the referenced literature lists of included publications. This resulted in a total of 14 studies corresponding to the 22 experiments performed in these studies, which were used in the qualitative study synthesis (Fig. 1). A subset of 13 studies (20 experiments) was used in the meta-analysis, as one experiment did not report SD/SEM, while the other did not report animal group sizes. All 13 of the included studies did not provide some or all numerical data required for meta-analysis in their respective manuscript texts, but they provided only graphical representations of their results. After the authors of these studies were contacted, 3 provided the numerical data, and for the rest of the studies digitization of the published graphs into the numerical data was done as detailed in the methods section.

### Animal characteristics

All 14 studies reported the animal species and strain; 9 (64%) used rats and 5 (36%) used mice. In studies that used rats, the predominantly used strain was Sprague–Dawley rats (7/9, 78%), while Long Evans and Lewis rats were both used once, respectively [(1 + 1)/9, 22%]. All studies using mice used the C57BL/6 strain. Regarding animal sex, all studies used male animals, although one used both male and female animals. Age was not declared in half (7/14) of the studies, and when declared either young adults (2–3 months for mice—3/4, 75%; 2–6 months for rats—2/3, 67%) or adults (3–16 months for mice—1/4, 25%; 6–20 months for rats—1/3, 33%) were used. Overall, the preferred choice of animals was young male adults of Sprague–Dawley rats or C57BL/6 mice.

### Stroke and splenectomy characteristics

In 13 (93%) of the 14 included studies, the applied model of ischemic stroke was middle cerebral artery occlusion (MCAO). The remaining study—conducted on mice—used a Rose Bengal dye photothrombotic stroke model, with lesions produced in cortical areas on the right side of the scalp, rostral to the bregma. Of the 13 studies using the MCAO model, 9 (69%) used a transient occlusion with ischemia duration ranging from 30 to 120 min (60 min being the most common), while the remaining 4 studies—all on Sprague–Dawley rats—used a permanent occlusion. Additionally, 4 (31%) studies using MCAO (3 transient and 1 permanent MCAO) did not report whether cerebral ischemia was confirmed with blood flow measurements. A total of 9 (64%) studies performed splenectomy 2 weeks prior to stroke induction (6 in rats and 3 in mice), 4 (29%) immediately prior to or after stroke (2 in mouse and rats each), and one study showed results with splenectomy performed immediately after stroke induction as well as 3 days after stroke (using rats). Overall, the most frequently used experimental model was a combination of permanent MCAO in Sprague–Dawley rats with splenectomy performed 2 weeks before, used by 4 studies (29%).

### Risk of bias in included studies

**Fig. 2:**
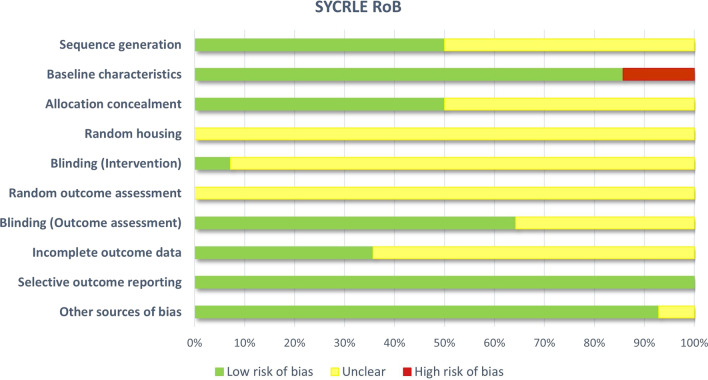
Risk of bias evaluated using the SYRCLE risk of bias (RoB) tool for animal studies

**Fig. 3:**
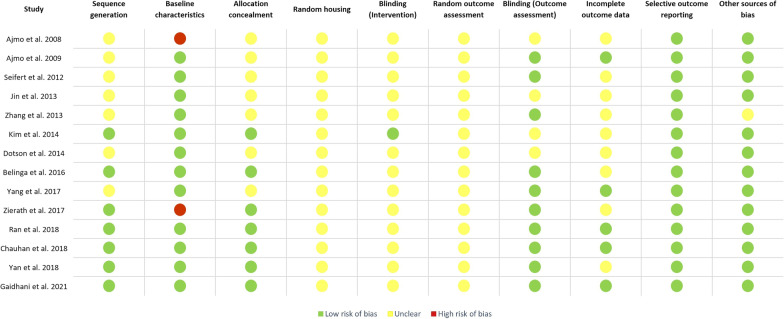
Risk of bias evaluation results presented in the form of a traffic-light plot. Risk of bias was evaluated using the SYRCLE risk of bias (RoB) tool for animal studies

All 14 studies included in the qualitative synthesis were evaluated using the SYRCLE risk of bias tool for animal studies [14] (Figs. 2 and 3). Half of the studies (7/14) reported adequate animal allocation sequence generation and application, and adequate concealment of animal group allocation. None of the studies reported if animals or cages were randomly placed in the housing facility, and whether animals had been chosen randomly for outcome assessment. Blinding for outcome assessment was reported in 9 (64%) studies, while only one study explicitly reported blinding of caregivers and/or investigators to splenectomy status. Incomplete outcome data such as missing or excluded animals were adequately addressed in 5 (36%) studies. All studies reported their respective expected outcomes and were determined to be free of selective outcome reporting. Information regarding other probable sources of bias (e.g., funding sources, animal welfare regulations, etc.) was reported in all but one study.

### Outcome measurement methods

#### Infarct volume

All 14 included studies measured infarct volume, albeit using different methodologies and at varying time points. The most frequently used method—employed by 5 studies—was 2,3,5-triphenyltetrazolium chloride (TTC) staining of coronal brain sections and subsequent infarct volume measurement using image analysis software. Other methods used were as follows: Nissl staining (3 studies), Fluoro-Jade + Nissl staining (2 studies), Fluoro-Jade staining (1 study), H&E staining (1 study), cryosectioning (although the staining method used was not declared; 1 study), and MRI evaluation (1 study). The time points at which infarct volume was measured ranged from 1 day to 1 month, with the most frequent time point being 4 days after stroke induction (in 5 out of 14 studies).

#### Neurological deficit scores

The seven studies measuring neurological deficit scores all used separate neurological deficit scales; the maximal neurological deficit score ranged from 4 to 28 between employed scales. Additionally, behavioral outcomes were measured at different time points and to account for this fact, only the last reported time point of assessment was used in the meta-analysis. This ranged from 4 to 28 days after stroke induction, with the latter being the most frequent.

### Meta-analysis

Meta-analysis was performed according to reported infarct volume data and neurological deficit scores. Adequate data for both outcomes were reported in 5 studies, for infarct volume alone in 7 studies, and for neurological deficit scores alone in only 1 study. In other words, infarct volume was analyzed in 12 studies, and neurological scores in 6. The meta-analysis of infarct volumes used a three-level random-effects meta-analysis model, while the analysis of neurological deficit scores used a conventional two-level random-effects meta-analysis model.

#### Infarct volume

**Fig. 4:**
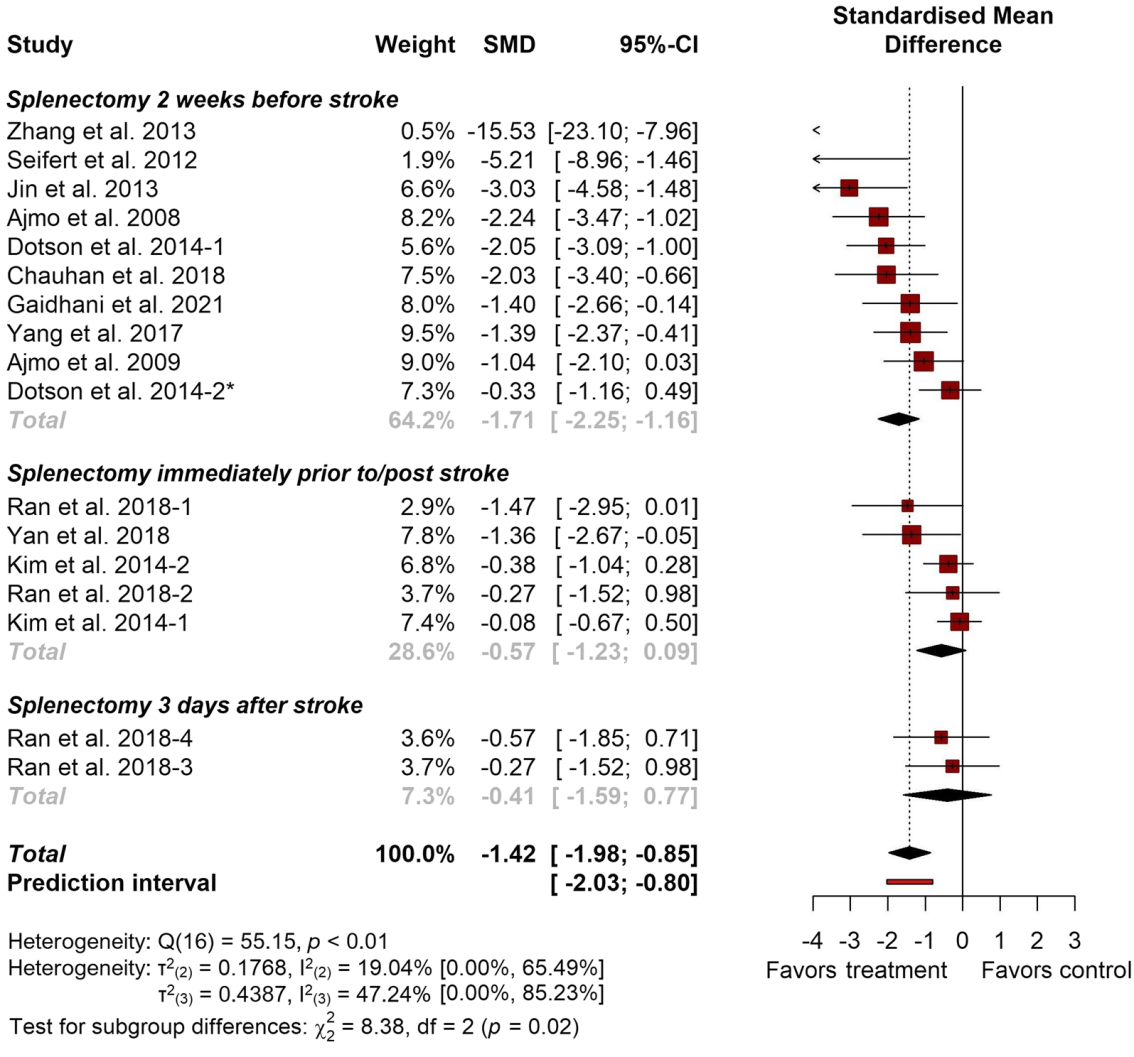
Forest plot detailing the overall effect of splenectomy on infarct volume. Study effect sizes are presented as standardized mean differences (SMD) along with their respective 95% confidence intervals. *Only experiment using female animals

The pooled data of 12 studies (corresponding to 17 experiments) that reported infarct volumes showed a reduction in splenectomized animals compared to the control groups (− 1.42; 95% CI [− 1.98, − 0.85]; 95% PI [− 2.03, − 0.80]) (Fig. 4). The estimated heterogeneity variance components were τ^2^_(2)_ = 0.1768 [0.0000; 1.4271], and τ^2^_(3)_ = 0.4387 [0.0000; 2.8272]. This equates to *I*^2^_(2)_ = 19.04% [0.00%, 65.49%] of the total variance being attributed to within-study (cluster) heterogeneity and *I*^2^_(3)_ = 47.24% [0.00%, 85.23%] to between-study heterogeneity. The three-level model did not provide a statistically significant better fit than a traditional two-level model with τ^2^_(3)_ set to zero (χ^2^_1_ = 16.10; *p* = *0*.2235), however, due to the inherent dependency of effect sizes within studies it is expected to provide a more theoretically adequate model for the analyzed data. Additionally, a basic outlier removal with effect size recalculation was conducted based on no overlapping confidence intervals with the overall effect size. This showed no major effect size differences, but a notable reduction in both within- and between-study heterogeneity (Table 1).

**Table 1:** Results of the meta-analysis with and without outlier removal based on no overlapping confidence intervals with the overall effect size

Analysis	SMD	95% CI	*p*	*I*^2^_(2)_	*I*^2^_(3)_
Main analysis	− 1.42	− 1.98, − 0.85	< 0.0001	19.04% [0.00%, 65.49%]	47.24% [0.00%, 85.23%]
Outliers removed*	− 1.30	− 1.77, − 0.82	< 0.0001	13.99% [0.00%, 69.52%]	40.43% [0.00%, 76.43%]

*Removed as outliers: “Zhang et al. 2013”, “Kim et al. 2014-1”

In a further attempt to understand the reasons for the observed heterogeneity, subgroup analysis was conducted based on study splenectomy timing relative to stroke induction. Overall subgroup effect sizes were as follows: splenectomy performed 2 weeks before (− 1.71; 95% CI [− 2.25, − 1,16], *Z* = − 6.1358, *p* ≤ 0.0001), splenectomy performed immediately prior to/post stroke (− 0.57; 95% CI [− 1.23, 0.09], *Z* = − 1.6867, *p* = 0.0917) and splenectomy performed 3 days after stroke (− 0.41; 95% CI [− 1.59, 0.77], *Z* = − 0.6779, *p* = 0.4979). The heterogeneity variances at level 2 and level 3 were τ^2^_(2)_ = 0.2697 [0.0000; 1.3703], and τ^2^_(3)_ = 0.0191 [0.0000; 1.6140], respectively. Splenectomy timing explained *R*^2^_(2)_ = 0.00% of the within-study heterogeneity and *R*^2^_(3)_ = 95.64% of the between-study heterogeneity.

The observed effects differed significantly between subgroups (*p* = 0.02) with subgroup pairwise comparisons showing a significant difference between splenectomy performed 2 weeks prior to stroke and immediately prior to/post stroke or 3 days after stroke, respectively (Table 2).

**Table 2:** Results of the pairwise subgroup comparisons using a *Z*-test

Pairwise comparison	*Z*	*p*
Splenectomy 2 weeks before stroke vs splenectomy immediately prior to/post stroke	− 2.60	0.0093
Splenectomy 2 weeks before stroke vs splenectomy 3 days after stroke	− 1.96	0.0497
Splenectomy immediately prior to/post stroke vs splenectomy 3 days after stroke	− 0.23	0.8145

Taken together, these results indicate that splenectomy provided favorable effects on infarct volumes in murine models of ischemic stroke.

#### Neurological deficit scores

**Fig. 5:**
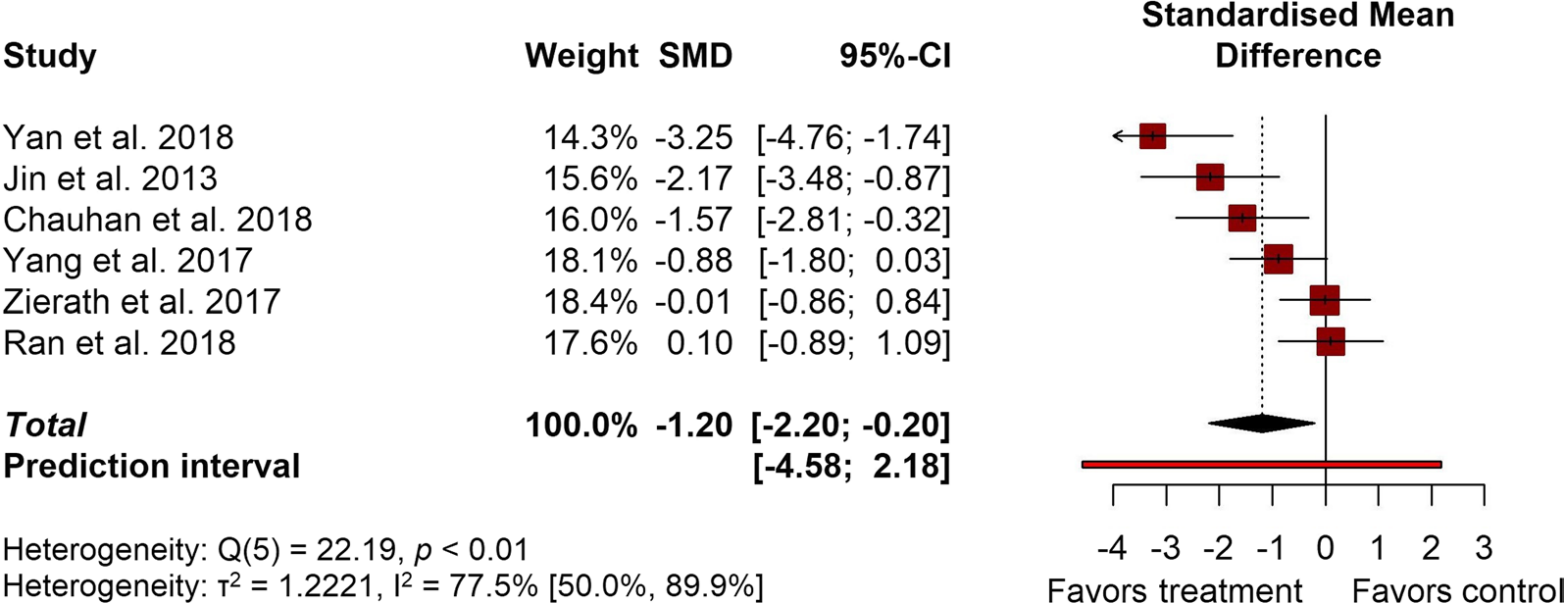
Forest plot detailing the overall effect of splenectomy on neurological deficit scores. Study effect sizes are presented as standardized mean differences (SMD) along with their respective 95% confidence intervals

A total of 6 studies (corresponding to 6 experiments) were included in the pooled effect size calculation for neurological deficit scores—one study (1 experiment) from the qualitative synthesis was not included due to no reported SD/SEM. Splenectomy was performed 2 weeks prior to stroke induction in one half of the experiments, and immediately prior to/after stroke in the other. As was the case with infarct volumes, the overall effect size for neurological deficit scores also showed an improvement in animals that had their spleen removed (− 1.20; 95% CI [− 2.20, − 0.20]; 95% PI [− 4.58, 2.18]) (Fig. 5). However, significant between-study heterogeneity was present (*I*^2^ = 77.5%; 95% CI [50.0%, 89.9%]). Due to the small number of studies reporting neurological deficit scores, no subgroup analysis was performed for this outcome. Similar to infarct volume analysis, these results point to a potential benefit of spleen removal for behavioral outcomes in murine models of ischemic stroke.

### Publication bias

**Fig. 6:**
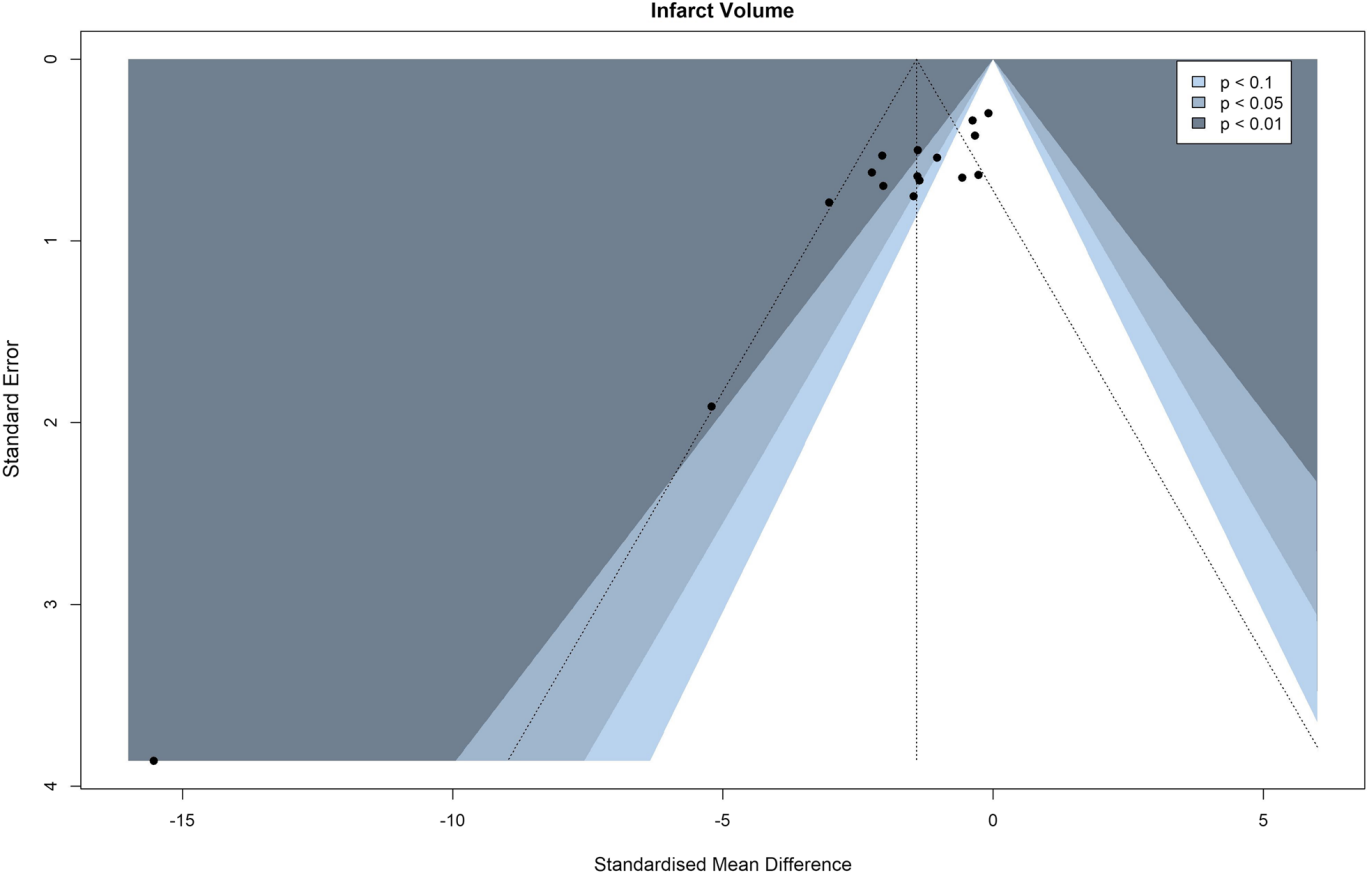
Funnel plot of infarct volume effect sizes

**Fig. 7:**
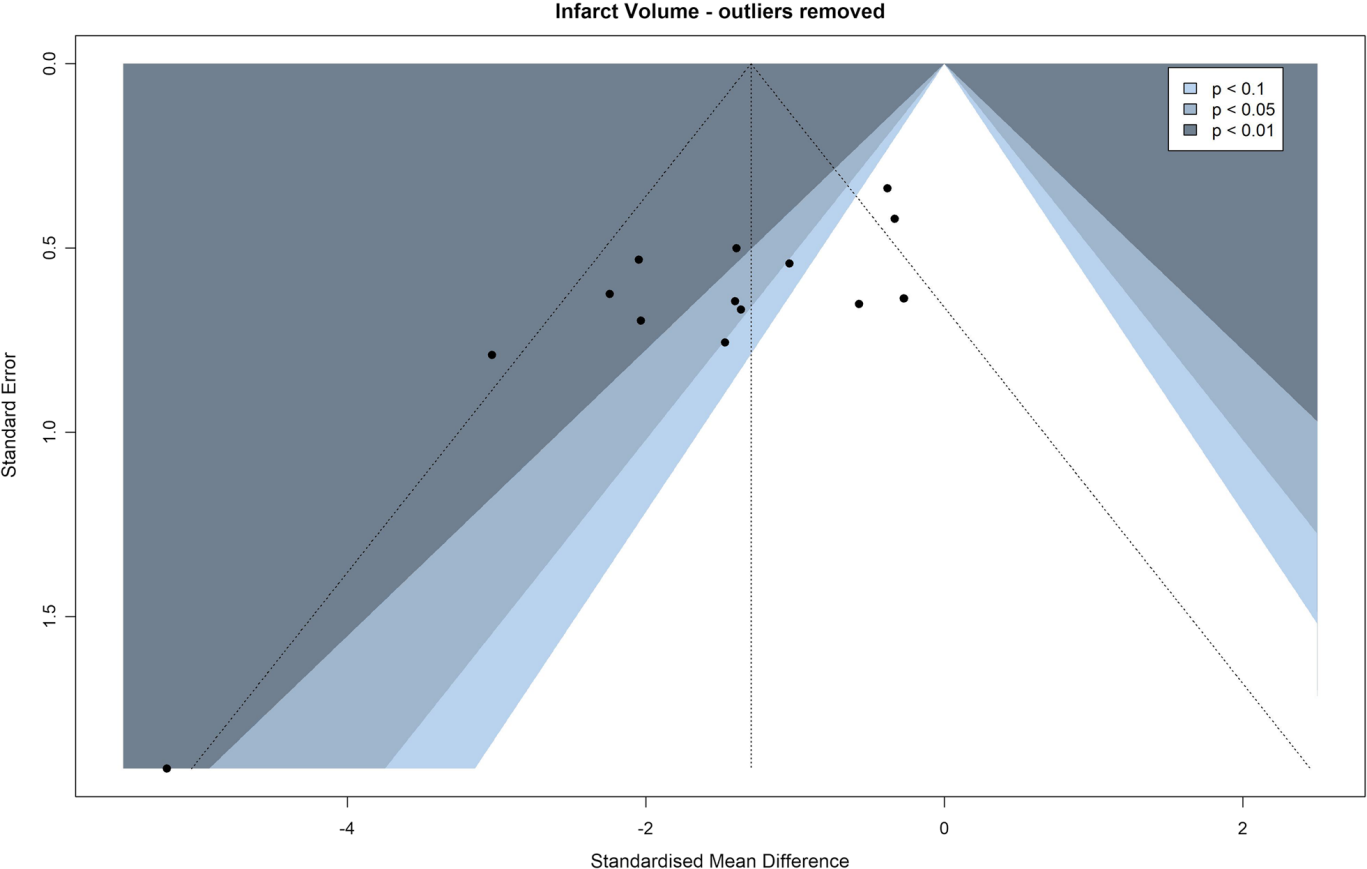
Funnel plot of infarct volume effect sizes with outliers removed based on no overlapping confidence intervals with the overall effect size. Removed as outliers: “Zhang et al. 2013”, “Kim et al. 2014–1”

**Fig. 8:**
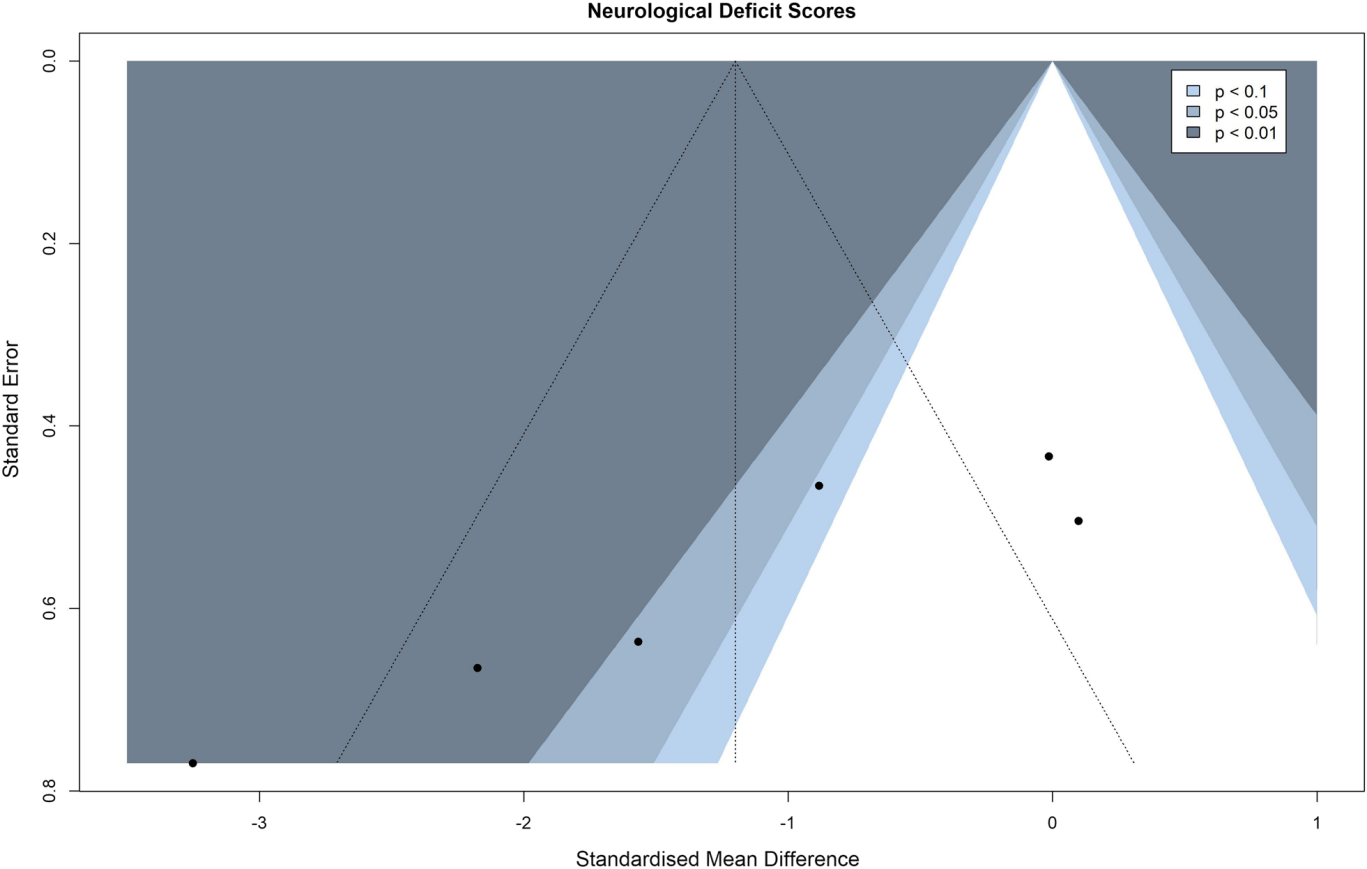
Funnel plot of neurological deficit score effect sizes

To evaluate publication bias, contour-enhanced funnel plots were produced for both primary outcome measures, including an additional funnel plot for infarct volumes with outliers removed as previously explained to better accommodate for the visual and statistical inspection of asymmetry (infarct volume—Figs. 6 and 7, neurological deficit scores—Fig. 8). In these plots, asymmetry was present and validated using Egger’s regression test (Table 3). Visual inspection of the infarct volume funnel plots revealed one potential missing study in the area of statistical non-significance (the potentially missing pair of “Seifert et al. 2012”, SMD = − 5.21). This could point to a potential presence of publication bias, however, other reasons such as observed heterogeneity, potentially lower methodological quality in smaller studies, or simple chance might be responsible for the funnel plot asymmetry. Visual inspection of the neurological deficit score funnel plot did not reveal any obvious missing studies in the areas of statistical non-significance, pointing to other potential reasons for asymmetry. Of the aforementioned reasons, simple chance can be considered a very probable cause due to the small number of studies reporting neurological deficit scores.

**Table 3:** Egger’s regression test results for infarct volumes, infarct volumes with outliers removed and neurological deficit scores

Egger’s regression test	Intercept value	95% CI	*t*	*p*
Infarct volume	− 3.717	− 5.05, − 2.38	− 5.47	< 0.0001
Infarct volume—outliers removed	− 3.226	− 5.20, − 2.38	− 3.20	0.0070
Neurological deficit scores	− 8.756	− 12.78, − 4.73	− 4.26	0.0130

### Cellular and molecular effects of splenectomy

In addition to infarct volumes and neurological deficit scores, multiple studies also analyzed the cellular and molecular markers of spleen removal in ischemic stroke. As expected, confirming the desired effect of the splenectomy, the levels of infiltrating immune cells in the infarct area, including neutrophils, monocytes/macrophages and T cells were reduced [8, 10, 21, 22]. Furthermore, splenectomy altered the expression of multiple inflammation-related cytokines in both the brain and blood serum. Two studies reported a significant reduction of IFN-γ levels in the brain at 72 h and 96 h after ischemia compared to sham-operated animals [9, 23]. Conversely, Dotson et al. reported an increase in IFN-γ production at the lesion site in both male and female animals 96 h after ischemia [24]. Another study reported that the levels of two other pro-inflammatory cytokines IL-1β and TNF-α were significantly reduced in the brains and sera of splenectomized animals, while the levels of IL-10—an anti-inflammatory cytokine—were higher [21]. Cellular apoptosis was also downregulated by splenectomy following stroke [25].

## Discussion

### Summary of findings

This qualitative systematic review and meta-analysis of splenectomy as preclinical intervention in ischemic stroke conducted on 14 selected studies showed that the preferred features of the preclinical intervention were the use of Sprague–Dawley rats, young males, and splenectomy performed 2 weeks before transient MCAO. Regarding the evaluation of the achieved effect, all studies measured the infarct volume and half of the studies reported neurological scores. However, the approaches to measure these outcomes were rather variable. Most of the studies used brain sections, applying diverse staining methods, and only one used MRI to assess the infarct volume. A similar situation was observed with behavioral outcomes; every study measuring neurological scores used a different deficit scale. The meta-analysis of these two outcomes (infarct volume and neurological deficit scores) favored splenectomy; however, these results should be taken with caution due to statistically significant heterogeneity and a potential presence of publication bias.

### Splenectomy effect size and heterogeneity analysis

For standardized mean differences, effect size magnitudes of > 0.2 are typically categorized as small, > 0.5 as moderate, and > 0.8 as large [26]. In this regard, the results of this meta-analysis showed that the pooled effect sizes for structural (infarct volume, − 1.42; 95% CI [− 1.98, − 0.85]; 95% PI [− 2.03, − 0.80]) and behavioral outcomes (neurological deficit scores, (− 1.20; 95% CI [− 2.20, − 0.20]; 95% PI [− 4.58, 2.18]) could be classified as large, strongly favoring splenectomy. When compared to the other recent meta-analysis of preclinical stroke models, interventions using stem cells, extracellular vesicles and virus-mediated gene transfer showed comparably beneficial effects, all to be classified as large [27–30].

The large effect size obtained was in contrast with other parameters considered, including study risk of bias, between-study heterogeneity, and funnel plot asymmetry. In particular, because substantial heterogeneity was present in both primary outcome analyses, the effect size results of this rather diverse group of experiments should be used for orientational purposes only. As a potential source of heterogeneity and effect size differences, splenectomy timing was considered in an additional subgroup analysis of infarct volumes, demonstrating that earlier spleen removal, 2 weeks prior to stroke induction, had significantly better outcomes when compared to splenectomy performed immediately prior to or after ischemia induction (*p* = 0.0093, *p* = 0.0497). However, even though the effect sizes of splenectomy performed at later time points—coinciding with the ischemic lesion or immediately after—could be classified as moderate or low, respectively, these differences cannot be disregarded, as they provide data more appropriately aligned with the time windows of potential ischemic stroke therapies. It should be noted that the observed heterogeneity is also based on many differences among included studies, most notably the various approaches to outcome measurement which differed in both timing and methodology. It would be ideal to better understand the sources of this variability—what is attributable to the outcome measurement method and what to the effect of interest, and how this relates to the intrinsic variability of clinical stroke.

### Study limitations

This study only investigated the overall effects of splenectomy in healthy adult mice or rats, while other animal models of ischemic stroke were not considered. Due to this limitation, the sample size of included studies is quite small, especially when considering the number of studies reporting neurological deficit scores.

Additional limitations of this study are further grounded in the limitations of the included studies themselves; most studies did not report an extensive amount of appropriate information related to possible risk of bias in their experiments, with some categories such as the random housing of animals and their selection for outcome assessment being reported by no studies at all. Furthermore, no individual study had adequately met all the criteria included in the risk of bias evaluation tool. Moreover, all included studies did not report some or all numerical data required for meta-analysis directly in their respective manuscripts. After we obtained the original data for 3 studies by a direct request to the authors, the use of digitization methods was needed to transform the visual data from the published figures to the corresponding numbers. Although this was done by two investigators independently, it can be considered a minor limitation due to the intrinsic nature of human imprecision.

When conducting the meta-analysis of infarct volumes, several effect sizes were identified as outliers based on no overlapping confidence intervals with the overall effect size. This was followed by an investigation of the respective studies, however, no obvious methodological differences were found that could be cause for these outlying effect sizes.

Lastly, another limitation of the analyzed studies was a potential presence of publication bias. Publication bias could be considered as a plausible explanation of infarct volume funnel plot asymmetry when accounting for the potentially missing pair of one effect size in the non-significance region. However, this asymmetry could also be caused by other reasons including heterogeneity, methodological quality and simple chance—the latter being especially true when observing the funnel plot of neurological deficit scores, due to the small number of studies reporting these measurements. Additionally, it is obvious that studies with higher standard errors reported higher effect sizes and vice versa, that the studies with the lowest standard errors reported very little to no benefits of splenectomy. Consequently, a reasonable conclusion can be drawn that internal (study quality and risk of bias) and external validity (publication bias) of future studies should be improved upon to achieve more robust and trustworthy results. It is therefore recommended that all future preclinical studies diligently adhere to up-to-date guidelines (e.g., ARRIVE guidelines [31]) on good experimental and reporting practices.

### Future translational potential of the obtained results

Despite all caveats, the beneficial pooled effect size of splenectomy points to its translational potential and indicates that it should be explored further as a therapeutical option in ischemic stroke. However, the idea of splenectomy being performed prophylactically as general approach or even in very specific high-risk cases is difficult to envisage as a viable therapeutic strategy due to its known side effects [32]. Moreover, a large study on patients who underwent splenectomy previously in their lives due to splenic injury showed an increased risk of both hemorrhagic and ischemic stroke [33].

One of the key issues of splenectomy as a preclinical intervention is clarifying the mechanism of translationally relevant improvements. The analyzed studies included a cellular and molecular characterization of splenectomy effects and confirmed that together with infiltrating monocytes, the number of accumulating neutrophils and T cells was also reduced in the infarct area. However, a detailed molecular sequence of these events still needs to be elucidated. Moreover, the complexity of the time-dependent evolution of post-stroke damage and repair would require studies following the outcomes through time, with a hope to recognize and differentiate the neuroprotective and neurorestorative effects [34, 35].

Splenectomy is not the only viable technique of achieving functional asplenia. One example of this is an experiment by Ostrowski et al., which showed that splenocyte apoptosis induced by acute splenic irradiation after MCAO significantly reduced infarct volumes and invading cell counts in Sprague–Dawley rats [36]. It could be envisaged that the same general principle of functional spleen removal may be achieved using less invasive and harmful methods, while selectively concentrating on the monocyte population. In this context, monocyte transmodulation was suggested as a promising novel approach in the therapy of ischemic stroke (recently reviewed in Park et al. 2020 [37]). While an accumulation of the monocytes in the brain via the locally expressed monocyte chemoattractant protein 1 (MCP-1) was associated with further injury exacerbation [38, 39], their selective depletion was shown to cause delayed clinical deterioration and hemorrhagic conversion of the infarct [40].

Furthermore, as both the pro-inflammatory Ly6C^high^ and anti-inflammatory Ly6C^low^ monocyte subtype have been shown to mobilize from the spleen and accumulate in the brain following ischemic stroke, novel strategies focused on depleting only specific spleen cell populations may be a more viable approach of determining their respective roles in the pathogenesis of ischemic stroke [10]. Of course, these experiments should not be limited only to monocytes/macrophages, as the spleen contains an abundant variety of other immunological cell populations which may be responsible for the benefits of splenectomy in ischemic stroke. A valid example to take into consideration are neutrophils, whose global selective depletion was shown to reduce blood–brain barrier breakdown and improve neovascularization during post-stroke recovery [41].

## Conclusions

This qualitative and quantitative systematic analysis of splenectomy as preclinical intervention in ischemic stroke showed its beneficial effects, which could be classified as large. Despite the mentioned limitations related to the presence of risks of bias, heterogeneity of the studies and potential publication bias, the spleen and its functional cell populations appear as promising potential targets for therapeutic modulation of the inflammatory response after ischemic stroke.

## Supplementary Information

**Additional file 1. **Reproducible database searches and the results obtained.

**Additional file 2: Table S1. **Data extracted for qualitative synthesis from 14 selected studies on splenectomy as preclinical intervention in murine models of ischemic stroke.

**Additional file 3: Table S2. **Data extracted for quantitative synthesis (meta-analysis) from 13 selected studies on splenectomy as preclinical intervention in murine models of ischemic stroke.

**Additional file 4: Table S3. **Risk of bias evaluation results from the 14 selected studies on splenectomy as a preclinical intervention in murine models of ischemic stroke.

## Data Availability

All data generated or analyzed during this study are included in this published article and are given as Additional file Tables S1, S2 and S3.

## Abbreviations

CCR2: C–C chemokine receptor 2
CI: Confidence interval
CX3CR1: CX3C chemokine receptor 1
H&E: Hematoxylin and eosin
IFN-γ: Interferon-γ
IL-1β: Interleukin-1β
IL-10: Interleukin-10
Ly6C: Lymphocyte antigen 6C
MCAO: Middle cerebral artery occlusion
MCP-1: Monocyte chemoattractant protein 1
MRI: Magnetic resonance imaging
PL method: Profile likelihood method
PI: Prediction interval
REML estimator: Restricted maximum-likelihood estimator
RoB: Risk of bias
SD: Standard deviation
SEM: Standard error of the mean
SMD: Standardized mean difference
TNF-α: Tumor necrosis factor-α
TTC: Triphenyltetrazolium chloride

## References

[1] Feigin V, Stark B, Johnson C, Roth G, Bisignano C, Abady G (2021). Global, regional, and national burden of stroke and its risk factors, 1990–2019: a systematic analysis for the Global Burden of Disease Study 2019. Lancet Neurol.

[2] Powers W, Rabinstein A, Ackerson T, Adeoye O, Bambakidis N, Becker K (2019). Guidelines for the Early Management of Patients With Acute Ischemic Stroke: 2019 Update to the 2018 Guidelines for the Early Management of Acute Ischemic Stroke: a Guideline for Healthcare Professionals From the American Heart Association/American Stroke Association. Stroke.

[3] Jayaraj R, Azimullah S, Beiram R, Jalal F, Rosenberg G. Neuroinflammation: friend and foe for ischemic stroke. J Neuroinflammation. 2019;16(1). 10.1186/s12974-019-1516-2 PMC6617684 31291966

[4] Jin R, Yang G, Li G (2010). Inflammatory mechanisms in ischemic stroke: role of inflammatory cells. J Leukoc Biol.

[5] Breckwoldt M, Chen J, Stangenberg L, Aikawa E, Rodriguez E, Qiu S (2008). Tracking the inflammatory response in stroke in vivo by sensing the enzyme myeloperoxidase. Proc Natl Acad Sci.

[6] Kratofil R, Kubes P, Deniset J (2017). Monocyte conversion during inflammation and injury. Arterioscler Thromb Vasc Biol.

[7] Swirski F, Nahrendorf M, Etzrodt M, Wildgruber M, Cortez-Retamozo V, Panizzi P (2009). Identification of splenic reservoir monocytes and their deployment to inflammatory sites. Science.

[8] Ajmo C, Vernon D, Collier L, Hall A, Garbuzova-Davis S, Willing A (2008). The spleen contributes to stroke-induced neurodegeneration. J Neurosci Res.

[9] Jin R, Zhu X, Liu L, Nanda A, Granger D, Li G (2013). Simvastatin attenuates stroke-induced splenic atrophy and lung susceptibility to spontaneous bacterial infection in mice. Stroke.

[10] Kim E, Yang J, Beltran C, Cho S (2014). Role of spleen-derived monocytes/macrophages in acute ischemic brain injury. J Cereb Blood Flow Metab.

[11] Zierath D, Shen A, Stults A, Olmstead T, Becker K (2017). Splenectomy does not improve long-term outcome after stroke. Stroke.

[12] Page M, Moher D, Bossuyt P, Boutron I, Hoffmann T, Mulrow C (2021). PRISMA 2020 explanation and elaboration: updated guidance and exemplars for reporting systematic reviews. BMJ.

[13] Rohatgi A. WebPlotDigitizer, version 4.5. Available at: https://automeris.io/WebPlotDigitizer. Accessed: 21 November 2021.

[14] Hooijmans C, Rovers M, de Vries R, Leenaars M, Ritskes-Hoitinga M, Langendam M. SYRCLE’s risk of bias tool for animal studies. BMC Med Res Methodol. 2014;14(1). 10.1186/1471-2288-14-43 PMC4230647 24667063

[15] R Core Team (2021). R: A language and environment for statistical computing. R Foundation for Statistical Computing, Vienna, Austria. Available at: https://www.R-project.org/. Accessed 22 November 2021.

[16] RStudio Team (2021). RStudio: Integrated Development Environment for R. RStudio, PBC, Boston, MA. Available at: http://www.rstudio.com/. Accessed 22 November 2021.

[17] Balduzzi S, Rücker G, Schwarzer G (2019). How to perform a meta-analysis with R: a practical tutorial. Evid Based Ment Health.

[18] Cheung MWL (2015). Meta-analysis: a structural equation modeling approach.

[19] Borenstein M, Hedges L, Higgins J, Rothstein H (2009). Introduction to meta analysis.

[20] Higgins JPT, Thompson SG (2002). Quantifying heterogeneity in a meta-analysis. Stat Med.

[21] Zhang BJ, Men XJ, Lu ZQ, Li HY, Qiu W, Hu XQ (2013). Splenectomy protects experimental rats from cerebral damage after stroke due to anti-inflammatory effects. Chin Med J (Engl).

[22] Ran Y, Liu Z, Huang S, Shen J, Li F, Zhang W (2018). Splenectomy fails to provide long-term protection against ischemic stroke. Aging Dis.

[23] Seifert H, Leonardo C, Hall A, Rowe D, Collier L, Benkovic S (2012). The spleen contributes to stroke induced neurodegeneration through interferon gamma signaling. Metab Brain Dis.

[24] Dotson A, Wang J, Saugstad J, Murphy S, Offner H (2015). Splenectomy reduces infarct volume and neuroinflammation in male but not female mice in experimental stroke. J Neuroimmunol.

[25] Belinga V, Wu G, Yan F, Limbenga E (2016). Splenectomy following MCAO inhibits the TLR4–NF-κB signaling pathway and protects the brain from neurodegeneration in rats. J Neuroimmunol.

[26] Cohen J (1988). Statistical power analysis for the behavioral sciences.

[27] Huang H, Qian K, Han X, Li X, Zheng Y, Chen Z (2018). Intraparenchymal neural stem/progenitor cell transplantation for ischemic stroke animals: a meta-analysis and systematic review. Stem Cells Int.

[28] Vu Q, Xie K, Eckert M, Zhao W, Cramer SC (2014). Meta-analysis of preclinical studies of mesenchymal stromal cells for ischemic stroke. Neurology.

[29] Thomas JM, Cunningham CJ, Lawrence CB, Pinteaux E, Allan SM (2020). Therapeutic potential of extracellular vesicles in preclinical stroke models: a systematic review and Meta-analysis. BMJ Open Sci.

[30] Skukan L, Brezak M, Ister R, Klimaschewski L, Vojta A, Zoldoš V (2021). Lentivirus- or AAV-mediated gene therapy interventions in ischemic stroke: A systematic review of preclinical in vivo studies. J Cereb Blood Flow Metab.

[31] Percie du Sert N, Hurst V, Ahluwalia A, Alam S, Avey M, Baker M et al. The ARRIVE guidelines 2.0: Updated guidelines for reporting animal research. PLOS Biol. 2020;18(7). 10.1371/journal.pbio.3000410 PMC7360023 32663219

[32] Buzelé R, Barbier L, Sauvanet A, Fantin B (2016). Medical complications following splenectomy. J Visc Surg.

[33] Lin J, Lin C, Lin M, Lai C, Lin H, Yang C (2015). Increased risk of hemorrhagic and ischemic strokes in patients with splenic injury and splenectomy. Medicine.

[34] Gorup D, Škokić S, Kriz J, Gajović S. Tlr2 deficiency is associated with enhanced elements of neuronal repair and caspase 3 activation following brain ischemia. Sci Rep. 2019;9(1). 10.1038/s41598-019-39541-3 PMC6391535 30808918

[35] Glasnović A, O’Mara N, Kovačić N, Grčević D, Gajović S. RANK/RANKL/OPG Signaling in the brain: a systematic review of the literature. Front Neurol. 2020;11. 10.3389/fneur.2020.590480 PMC7710989 33329338

[36] Ostrowski R, Schulte R, Nie Y, Ling T, Lee T, Manaenko A (2012). Acute splenic irradiation reduces brain injury in the rat focal ischemic stroke model. Transl Stroke Res.

[37] Park J, Chang J, Kim J, Lee J. Monocyte transmodulation: the next novel therapeutic approach in overcoming ischemic stroke?. Front Neurol. 2020;11. 10.3389/fneur.2020.578003 PMC7642685 33193029

[38] Schilling M, Strecker J, Schäbitz W, Ringelstein E, Kiefer R (2009). Effects of monocyte chemoattractant protein 1 on blood-borne cell recruitment after transient focal cerebral ischemia in mice. Neuroscience.

[39] Chen Y, Hallenbeck J, Ruetzler C, Bol D, Thomas K, Berman N (2003). Overexpression of monocyte chemoattractant protein 1 in the brain exacerbates ischemic brain injury and is associated with recruitment of inflammatory cells. J Cereb Blood Flow Metab.

[40] Gliem M, Mausberg A, Lee J, Simiantonakis I, van Rooijen N, Hartung H (2012). Macrophages prevent hemorrhagic infarct transformation in murine stroke models. Ann Neurol.

[41] Kang L, Yu H, Yang X, Zhu Y, Bai X, Wang R et al. Neutrophil extracellular traps released by neutrophils impair revascularization and vascular remodeling after stroke. Nat Commun. 2020;11(1). 10.1038/s41467-020-16191-y PMC7237502 32427863

