# Supplementary material for: The effects of splenectomy in murine models of ischemic stroke: a systematic review and meta-analysis
Source: J Neuroinflammation. 2022 Sep 23;19:233. doi: 10.1186/s12974-022-02593-w (PMC9508771; doi:10.1186/s12974-022-02593-w)

**Literature searching**

| **Database** | **Hits** |
| --- | --- |
| PubMed | 25 |
| Web of Science | 46 |
| SCOPUS | 20 |

Database: **PubMed**

Date Searched: 28/6/2022

Search Strategy:

| **#** | **Search** | **Results** |
| --- | --- | --- |
| 1 | splenectomy | 31,435 |
| 2 | mouse OR rat OR mice OR rats OR murine | 3,698,947 |
| 3 | brain OR neuron OR neurons OR astrocytes OR glia OR neuroglia OR microglia | 2,623,909 |
| 4 | ischemia OR ischaemia OR ischemic lesion OR stroke | 816,246 |
| 5 | (((splenectomy) AND (mouse OR rat OR mice OR rats OR murine)) AND (brain OR neuron OR neurons OR astrocytes OR glia OR neuroglia OR microglia)) AND (ischemia OR ischaemia OR ischemic lesion OR stroke) | 25 |


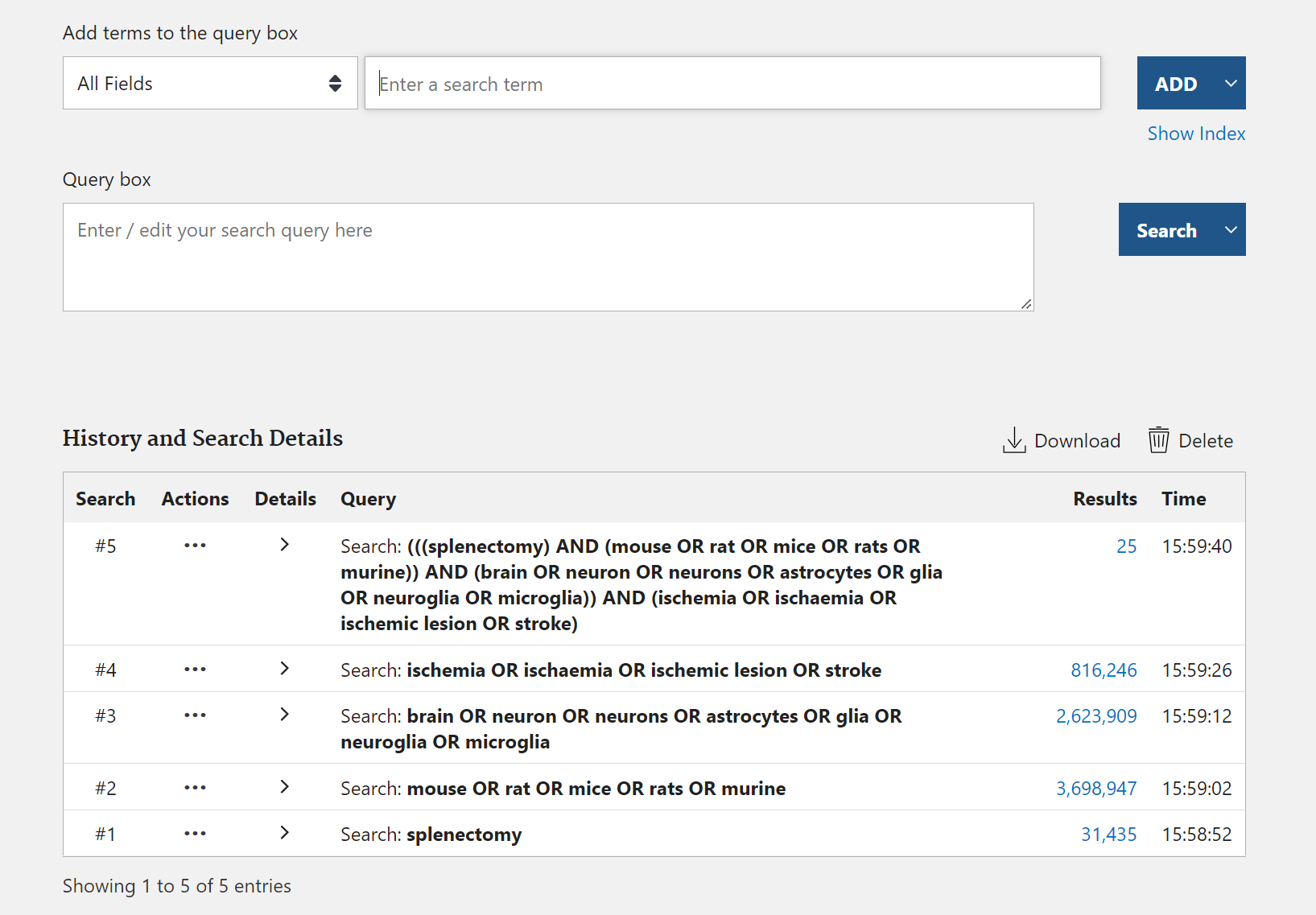


Database: **Web of Science**

Date Searched: 28/6/2022

Search Strategy:

| **#** | **Search** | **Results** |
| --- | --- | --- |
| 1 | TS=(splenectomy) | 41,537 |
| 2 | TS=(mouse OR rat OR mice OR rats OR murine) | 5,853,557 |
| 3 | TS=(brain OR neuron OR neurons OR astrocytes OR glia OR neuroglia OR microglia) | 3,150,153 |
| 4 | TS=(ischemia OR ischaemia OR ischemic lesion OR stroke) | 1,112,511 |
| 5 | #1 AND #2 AND #3 AND #4 | 46 |


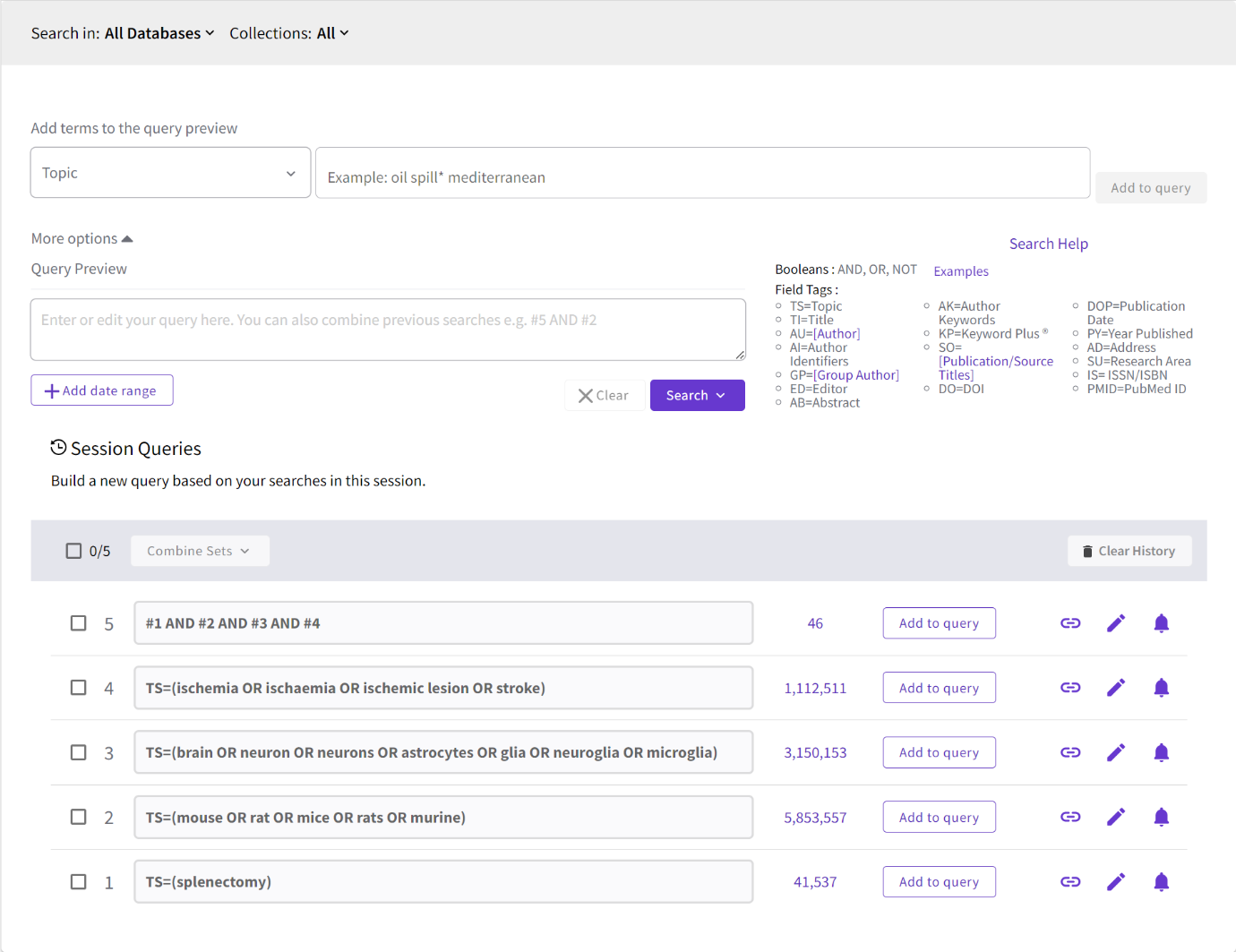


Database: **SCOPUS**

Date Searched: 28/6/2022

Search Strategy:

| **#** | **Search** | **Results** |
| --- | --- | --- |
| 1 | TITLE-ABS-KEY ( splenectomy ) | 44,408 |
| 2 | TITLE-ABS-KEY ( mouse OR rat OR mice OR rats OR murine ) | 4,142,777 |
| 3 | TITLE-ABS-KEY ( brain OR neuron OR neurons OR astrocytes OR glia OR neuroglia OR microglia ) | 2,821,975 |
| 4 | TITLE-ABS-KEY ( ischemia OR ischaemia OR ischemic lesion OR stroke ) | 186,213 |
| 5 | ( TITLE-ABS-KEY ( splenectomy )) AND ( TITLE-ABS-KEY ( mouse OR rat OR mice OR rats OR murine )) AND (TITLE-ABS-KEY ( brain OR neuron OR neurons OR astrocytes OR glia OR neuroglia OR microglia )) AND (TITLE-ABS-KEY ( ischemia OR ischaemia OR ischemic lesion OR stroke )) | 20 |


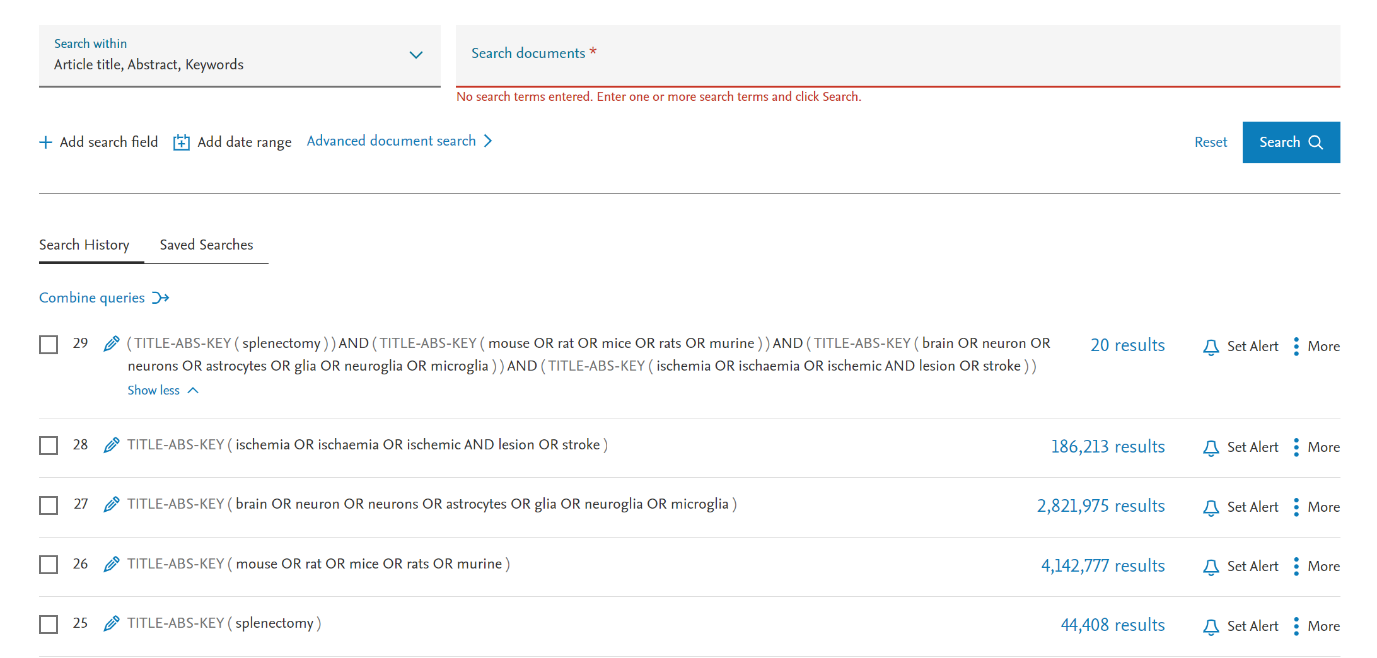

Supplement: Supplementary file 1 — Additional file 1. Reproducible database searches and the results obtained. [file 12974_2022_2593_MOESM1_ESM.docx]
